# Consecutive Moderate and Severe Drought Stresses Affect Chlorophyll Fluorescence and Non‐structural Carbohydrates Dynamics in Grapevine Leaves

**DOI:** 10.1111/ppl.70535

**Published:** 2025-09-21

**Authors:** Monica Canton, Francesco Mirone, Franco Meggio, Alessandro Pichierri, Valentino Casolo, Giovanni Battista Tornielli, Andrea Pitacco

**Affiliations:** ^1^ Department of Agronomy, Food, Natural Resources, Animals and Environment—DAFNAE University of Padova Legnaro Padova Italy; ^2^ Interdepartmental Research Centre for Viticulture and Enology—CIRVE University of Padova Conegliano Treviso Italy; ^3^ Department of Agricultural Food Environmental Animal Sciences University of Udine Udine Italy

**Keywords:** carbon isotope composition, drought stress, gas exchange, sugars, transpiration rate, *Vitis vinifera* L

## Abstract

Drought events represent a growing challenge for agriculture in the Mediterranean region, particularly for *Vitis vinifera* , a species with economic and cultural significance. This study evaluates the effects of two subsequent drought stress events on grapevine cv. Sauvignon blanc, combining physiological and biochemical approaches. The trial was conducted in a semi‐controlled tunnel from mid‐June to mid‐July 2024 on potted vines. Plants were divided into well‐watered and drought groups. Drought‐stressed vines underwent two drought cycles: the first reaching −1.3 MPa stem water potential, followed by rewatering, and the second with either moderate (−1.3 MPa) or severe (−2.5 MPa) drought before another rewatering. Stomatal conductance, chlorophyll fluorescence, gas exchange, and nonstructural carbohydrate levels were measured to assess physiological responses. Grape bunches were analyzed for C13/C12 isotope ratios at harvest. Significant physiological and biochemical differences were observed between the first moderate drought and both the second moderate and severe drought, highlighting distinct plant responses to water stress and rewatering. In the second cycle, vines subjected to severe drought showed significantly reduced photosynthetic efficiency compared to those under moderate drought, suggesting a decline in resilience. Chlorophyll fluorescence data indicated sustained photoinhibition after severe drought, while differences in nonstructural carbohydrate levels between treatments and across different times of the day revealed shifts in carbon metabolism. Carbon isotope composition confirmed the effect of double water stress. These results highlight the grapevines' capacity for physiological adaptation to repeated drought while also indicating the potential accumulation of negative effects if stress becomes excessive or prolonged.

## 1 Introduction

Grapevine ( *Vitis vinifera* L.) is one of the most economically important fruit crops worldwide (Alston and Sambucci 2019). It is cultivated across a wide range of climates, from the Mediterranean to temperate regions, where ongoing climate change is having a significant impact (Fraga et al. 2013; Lionello et al. 2014). Climate plays a pivotal role in grapevine growth and development (Santos et al. 2011; Van Leeuwen et al. 2004), with specific environmental conditions, such as precipitation and temperature, shaping key physiological and phenological processes (Magalhães 2008) as well as vine productivity and wine quality (Faralli et al. 2024; Rogiers et al. 2022; Van Leeuwen et al. 2017).

In the Mediterranean region, precipitation is a critical factor. Projections indicate that future decreases in rainfall, coupled with rising temperatures, are likely to exacerbate drought events, posing significant challenges to viticulture (Lionello et al. 2014; Pachauri and Meyer 2014; Sodini et al. 2023). According to the Intergovernmental Panel on Climate Change (IPCC), global surface temperatures have increased by 1.09°C between 1850–1906 and 2011–2020, with more pronounced warming over land (1.59°C) than over oceans (0.88°C). This warming trend, the fastest recorded over the last 2000 years, is expected to intensify drought conditions in traditional wine‐growing regions, increasing reliance on irrigation (IPCC 2023).

Wine‐producing regions are also located in temperate climates, with warm and dry summers typical of sun‐exposed coastal and continental regions. Currently, most wine‐growing regions in Europe are cultivated under rainfed conditions, making the vines susceptible to drought (Charrier et al. 2018). Water availability and stress have varying impacts on grapevine depending on the phenological stage, cultivar, and intended wine style (Deloire et al. 2004; Miras‐Avalos and Araujo 2021; Santos et al. 2020). Although the water deficit can hinder vine growth and decrease yield, moderate stress during specific phenological stages can improve grape and wine quality, as long as it does not reach extreme levels. This provides the basis for implementing deficit irrigation strategies (Chaves et al. 2010). To optimize water‐use efficiency, it is essential to understand the physiological constraints imposed by water deficit and to identify possible thresholds to avoid long‐term damage, such as hastened fruit ripening or reduced buds' fruitfulness in subsequent seasons (Dayer et al. 2020). Nonetheless, grapevine exhibits a notable capacity to tolerate moderate to severe water deficits (Gambetta et al. 2020). One of the earliest response strategies to decline in water potential is stomatal regulation, driven by increased local synthesis of abscisic acid (ABA; Tombesi et al. 2015). Stomatal closure reduces water loss through transpiration but also limits gas exchange, leading to an unavoidable decline in photosynthesis. Although the effects of drought on the physiological behavior of plants have been extensively studied, research on plant responses to recurrent drought events remains limited, particularly on perennial crops, which are highly vulnerable to climate variations as they are exposed to fluctuating weather conditions throughout the year (Arias et al. 2022; Keller 2023). In drought‐prone regions, trees and vines experience repeated stress, which may condition their physiological responses over time. Previous studies on grapevine have primarily focused on isolated stress events, even though vineyards are rarely exposed to single, discrete stress episodes. In recent years, few studies have considered repeated cycles of water stress (Benyahia et al. 2023; Hochberg, Bonel, et al. 2017; Hochberg, Windt, et al. 2017; Hochberg et al. 2023; Tombesi et al. 2018), and to the best of our knowledge, this study is the first to integrate multiple physiological and biochemical factors including leaf gas exchange, chlorophyll fluorescence, carbon‐13 isotopic composition, and non‐structural carbohydrates (NSCs) to investigate grapevine responses to subsequent drought stress. Notably, NSCs were measured dynamically, with samples collected four times throughout the day. This multifaceted approach was applied across two distinct cycles of water stress, providing a comprehensive understanding of grapevine responses under these conditions.

Our study investigates how two successive drought events affect grapevine physiology, hypothesizing that repeated exposure to water deficit—whether moderate or severe—negatively influences plant recovery capacity. We hypothesize that a second drought cycle, following an initial moderate stress, may further disrupt physiological balance and impair mechanisms of stress tolerance and resilience. To explore this, we aim to: (i) evaluate grapevine responses to repeated water deficits by integrating physiological parameters measured at different times of the day; (ii) analyze the daily dynamics of NSCs under repeated drought conditions; and (iii) assess the efficacy of chlorophyll fluorescence indices in detecting water stress in grapevines.

## 2 Material and Methods

### 2.1 Plant Material and Experimental Setup

Experiments were carried out on 8‐year‐old potted grapevines, *Vitis vinifera* cv. Sauvignon blanc (clone 108) grafted onto Kober 5 BB rootstock (K5BB; *V. berlandieri* × *V. riparia* ). The experiment was conducted in a semi‐controlled transparent plastic‐film tunnel (Botton et al. 2022; Meggio et al. 2020; Ruperti et al. 2019) at the Experimental Farm of the University of Padua “L. Toniolo” in Legnaro, northeast of Italy during summer 2024 from mid‐June to mid‐July. In January, during the winter season, 50 plants were transplanted from 10 L pots into 30 L containers, all equipped with a drip irrigation system. All vines were in very good condition, without any sign of degeneration. The substrate used had a medium‐coarse texture, high porosity, and moderate water retention capacity. It was composed of blonde and brown peat with a small percentage of pumice (commercially known as Terflor—Vulcan Estivo PF substrate). Each grapevine had two shoots trained to grow up to 1.80 m in height using a vertical shoot positioning system.

**FIGURE 1:**
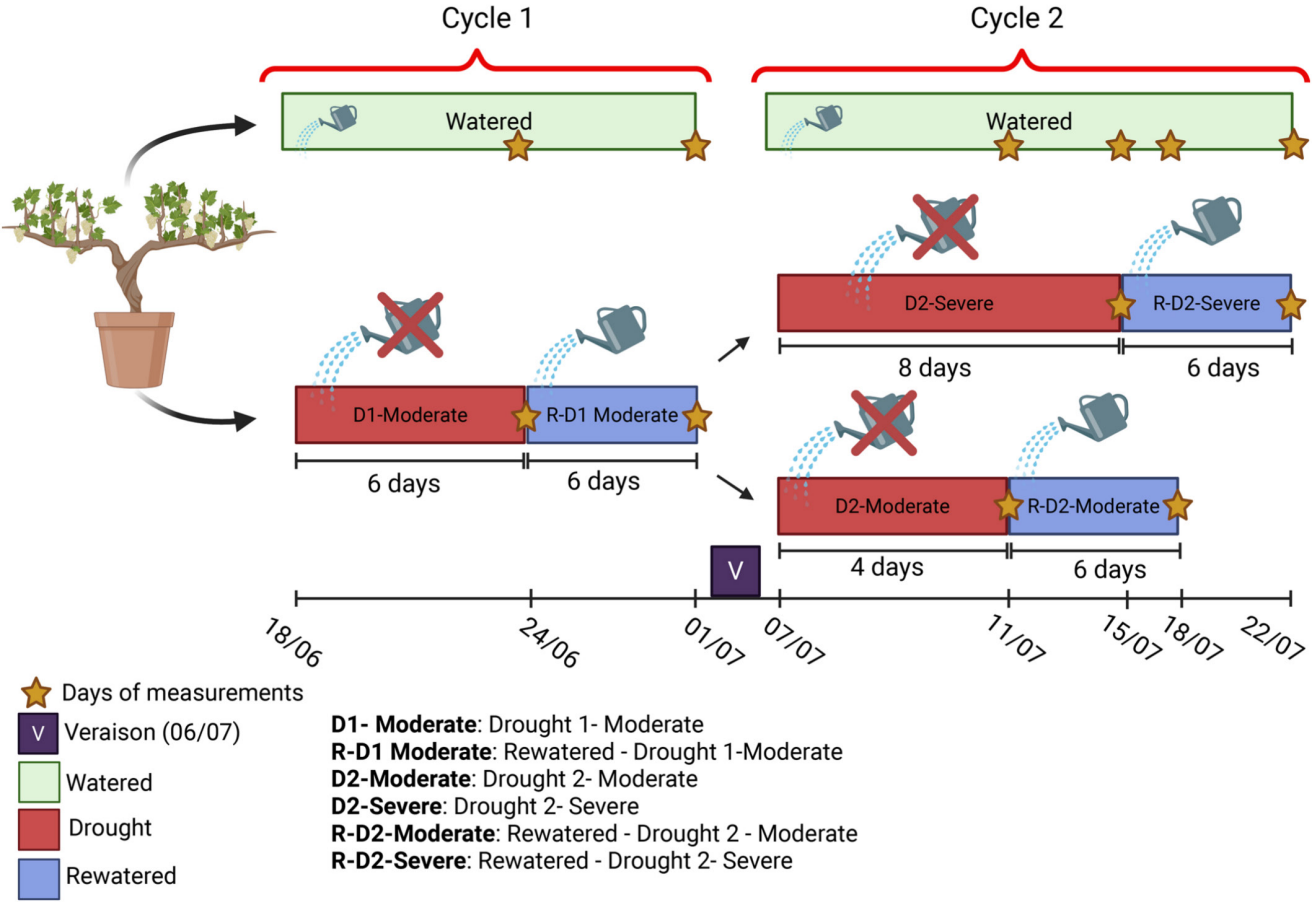
Experimental design with a timeline showing the key dates of the study. Watered plants, represented by a green bar, were continuously well‐watered throughout the experiment. The timeline includes the first moderate drought phase (Drought 1‐Moderate, red bar), followed by its rewatering phase (blue bar). Subsequently, the plants were divided into two groups: D2‐Moderate (second moderate drought) and D2‐Severe (severe drought) (red bars), each followed by their respective rewatering phases (R‐D2‐Moderate and R‐D2‐Severe, blue bars). Stars indicate the dates on which measurements were taken.

Within the experimental setup, two treatment groups were established, consisting of 20 watered plants and 30 plants exposed to drought conditions. The watered group was kept in well‐watered conditions (85%–90% of field capacity) throughout the experimental period, which started on the 18/06/2024 and continued until harvest on the 22/07/2024. On the other hand, for drought‐stressed plants, the water supply was stopped until reaching a stem water potential (Ψ_stem_) of −1.3 MPa (from now on: Drought 1‐Moderate, D1), which occurred after 6 days. This threshold indicates high water constraint conditions according to Van Leeuwen et al. (2009). Afterward, the plants exposed to drought conditions underwent a 6‐day rewatering period, during which they were maintained under watered conditions. After the rewatering phase (performed by irrigation), a second cycle of drought conditions was initiated. To implement this, the 30 plants exposed to drought conditions were further divided into two groups: 15 plants underwent a similar drought level to the D1 (Ψ_stem_ of −1.3 MPa), referred to hereafter as the Drought 2—moderate (D2‐Moderate), whereas the remaining 15 plants were exposed to a more severe drought (Ψ_stem_ of −2.5 MPa), referred to as the Drought 2—severe (D2‐Severe), indicating prolonged severe water constraint conditions according to Van Leeuwen et al. (2009). It took 4, 6, and 8 days, respectively, to reach these water potential levels. After both the D2‐Moderate and D2‐Severe droughts, the plants were again given a 6‐day rewatering period under well‐watered conditions (Figure 1). The water supply for each treatment was daily determined gravimetrically at 18:00 solar time, by weighing each pot using an electronic scale. Water was supplied twice a day, in the early morning (6:00 h) and in the evening (20:00 h).

Meteorological variables, such as air temperature (*T*_air_, °C), relative air humidity (RH, %), precipitation (*P*_tot_, mm), incoming global radiation (Rad, W m^−2^) and VPD (kPa) were recorded hourly by a weather station located within the tunnel (*T*_air_, RH, Rad) and by a complete weather station (*P*_tot_) located at less than 500 m from the experimental site (Botton et al. 2022).

### 2.2 Weather Conditions

**FIGURE 2:**
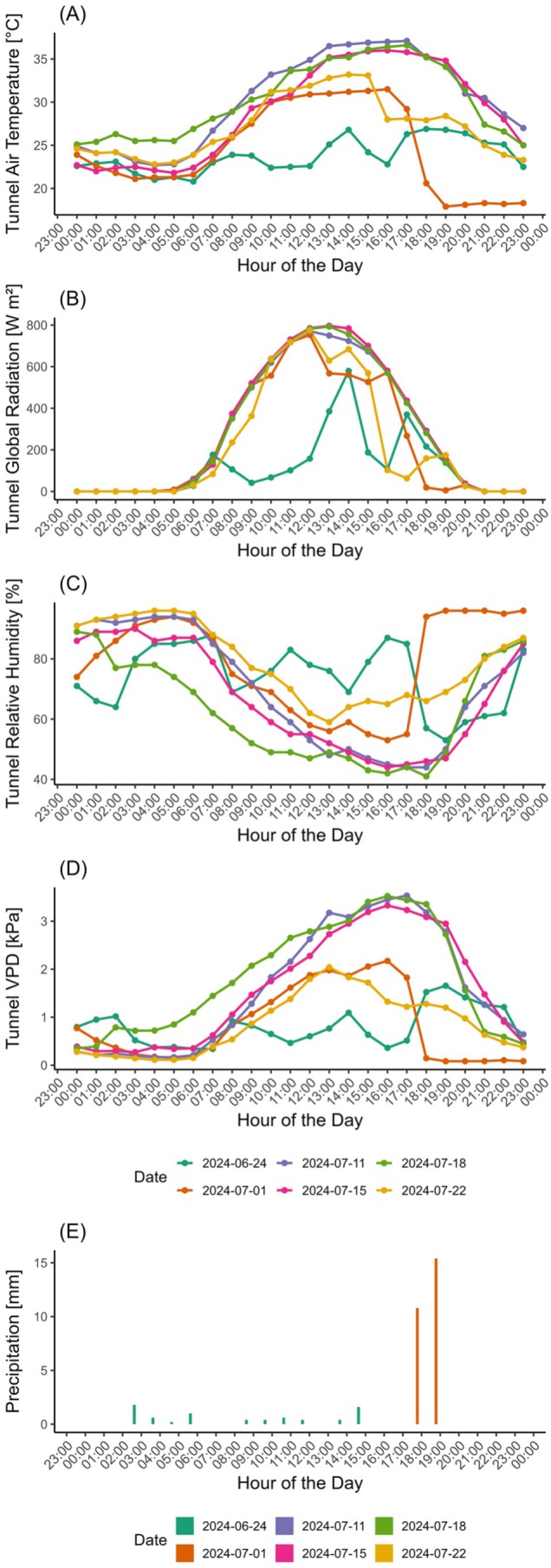
Hourly environmental conditions during the experiment. Data include (A) tunnel air temperature (°C), (B) tunnel global radiation (W m^−2^), (C) tunnel relative humidity (RH, %), (D) tunnel VPD and (E) precipitation (mm). The days correspond to the specific days of measurements.

The experimental period was predominantly marked by clear‐sky days, high temperatures, and elevated global solar radiation (Figure 2, Table S1). This pattern was consistent during the measurement days (Figure 1), except for the D1 event (24/06). On this specific day, precipitation occurred, resulting in a drop in temperatures and VPD that likely influenced vine physiology. During the measurement days, maximum and minimum air temperatures peaked at 37.1°C and 17.9°C, respectively. The maximum temperature difference between the external environment and the inside of the tunnel was 3.5°C. Detailed hourly data on outdoor conditions for June and July are provided in Table S1. The tunnel relative humidity (RH) remained between 40% and 60% during the hottest hours of the day.

### 2.3 Physiological Analyses

#### 2.3.1 Water Potential

The leaf water potential was measured at pre‐dawn (Ψ_pre‐dawn_, MPa) and midday (Ψ_midday_, MPa), as well as stem water potential (Ψ_stem_, MPa) using a Scholander‐type pressure chamber (model: PMS‐600, PMS Instruments). For each measurement, six randomly chosen, sun‐exposed, and fully expanded leaves per treatment were excised from the scions with a scalpel blade and immediately placed in the chamber. Ψ_pre‐dawn_ measurements were taken before sunrise, at approximately 04:00 h solar time. Ψ_midday_ and Ψ_stem_ measurements were conducted from 12:00 h to 13:00 h. To prevent transpiration and allow equilibration with the stem water potential, leaves used for Ψ_stem_ measurements were enclosed in opaque plastic bags for more than 1 h prior to excision.

#### 2.3.2 Sap Flow Measurements

The heat balance gauges used in this experiment were Dynamax (Model SGA‐10) stem flow gauges (Dynamax Inc.) to monitor sap flow rates in the grapevine plants. The sensors detect temperature differences above and below a heater and measure radial heat flux using thermocouple junctions placed around the stem. The gauges were deployed on three vines' replicates per treatment (watered, D2‐Moderate, and D2‐Severe droughts), selected upon homogeneity in the leaf area, and installed on grapevine stems, under the grafting point. Each heat balance gauge was fitted with foam covers, weather shields, and multiple layers of aluminum foil to reduce unwanted heat and radiative exchanges and ensure steady‐state energy transfer. Sap flow rate (g h^−1^) measurements were taken every 15 min and registered by a datalogger (CR1000X, Campbell Scientific).

#### 2.3.3 Gas Exchange and Chlorophyll Fluorescence

Single‐leaf gas exchanges and chlorophyll fluorescence were measured with an open‐system (LI‐6800, LI‐COR Inc.) at six dates: when plants reached the maximum drought and after 6 days of rewatering in each drought cycle (Figure 2). Measurements were conducted using a 6 cm^2^ leaf cuvette equipped with a Multiphase Flash Fluorometer (6800‐01A). Each leaf was subjected to at least 2 h of dark adaptation, using leaf clips provided by the manufacturer, and the Fv/Fm parameter was measured. After the dark adaptation, leaves were subjected to at least 20 min of acclimation at a CO_2_ concentration of 400 μmol CO_2_ mol^−1^ and a constant saturating photosynthetic photon flux density (PPFD) of 1500 μmol of photons m^−2^ s^−1^ before starting the measurements. Leaf temperature was maintained at 30°C, with RH ranging between 50% and 70%, resulting in a vapor pressure deficit (VPD) of approximately 1.5 kPa inside the chamber. Measurements were performed on at least three fully expanded leaves per treatment, between 11:00 h and 14:00 h solar time. Prior to the beginning of the measurements at 11:00 h, leaves had already been dark‐adapted for at least 2 h to ensure accurate determination of the Fv/Fm parameters. In addition, stomatal conductance was monitored daily on all experimental replicates using a leaf porometer/fluorometer (LI‐600, LI‐COR Inc.).

#### 2.3.4 Non‐structural Carbohydrate Quantifications

The analysis of NSCs was conducted on leaves sampled at the end of all drought‐rewatering cycles. Sampling was carried out four times during the day (3:00, 9:00, 15:00, and 21:00 h, solar time) to evaluate the dynamics of NSCs throughout the entire day. For each time point, three biological replicates were collected, each consisting of one leaf from three different plants (i.e., three leaves per replicate). Immediately after sampling, leaves were flash‐frozen in liquid nitrogen and subsequently stored at −80°C until analysis. The collected samples were microwaved at 600 W for 3 min to stop any enzymatic activity, dried, weighed, and then pulverized using a vibratory mill (Retsch model MM500 vario). For each sample, 15 ± 1 mg of powder, verified to be < 150 μm in size, was transferred to 2 mL Eppendorf tubes. NSC extraction followed the standardized protocol by Quentin et al. (2015) and Landhäusser et al. (2018), with modifications for small samples as proposed by Gargiulo et al. (2024).

##### 2.3.4.1 Soluble NSCs Extraction

Soluble NSCs, including monomers, dimers, and short oligomers, were extracted from the ground samples using ethanol. Each Eppendorf tube containing 15 ± 1 mg of sample received 500 μL of 80% ethanol (v/v), was vortexed, and incubated at 80°C for 30 min. After incubation, tubes were vortexed to homogenize the sample, then centrifuged at 22,000 g for 3 min (Hettich model MIKRO 120). The supernatant was collected and transferred to a 1.5 mL Eppendorf tube. This extraction was repeated with 300 μL of 80% ethanol (v/v), and the supernatant was pooled with the previously collected fraction. The 1.5 mL Eppendorf tubes containing the ethanol‐soluble NSCs were placed open in an oven at 55°C overnight to evaporate the ethanol. The next day, 500 μL of 50 mM Tris–HCl (pH 7.5) was added to the crystallized solutes in the tubes, which were then vortexed and stored at −20°C. The extraction of water‐soluble NSCs (including glucans and branched glucans) followed from the residual pellet in the 2 mL Eppendorf tubes after ethanol extraction. To each tube, 500 μL of deionized water was added, vortexed, and left to rest at room temperature for 24 h. After resting, the tubes were centrifuged at 22,000 g, and the supernatant containing the water‐soluble NSCs was transferred to a new 1.5 mL Eppendorf. The analysis of ethanol‐ and water‐soluble NSCs was performed using the anthrone assay by (Yemm and Willis 1954). Samples were prepared on a heat‐resistant Victor microplate. Each well received 5 μL of ethanol‐soluble sample or 10 μL of water‐soluble sample, 45 μL of distilled water, and 250 μL of sulfuric acid with 0.1% (m/v) anthrone. The plate was cooled on ice for 10 min, then heated at 100°C for 20 min. After cooling to room temperature for 10 min, the absorbance was measured using a spectrophotometer (PerkinElmer model Victor3 Multilabel Counter 1420) at 620 nm, corresponding to the anthrone absorption peak. Absorbance values were converted to glucose concentrations using a calibration curve prepared with glucose standards (0–0.1–0.2–0.5–1–2–4 mg mL^−1^).

##### 2.3.4.2 Starch Quantification

For starch analysis, 400 μL of 10 mM Tris–HCl (pH 6.9) was added to the residual pellet in the Eppendorf tubes from the water‐soluble NSC extraction. Samples were incubated at 100°C for 1 h to promote starch gelatinization. Afterwards, each sample received 100 μL of 10 mM Tris–HCl (pH 6.9) containing 100 U of α‐amylase, followed by incubation at 55°C overnight to enhance enzymatic activity. The next day, 500 μL of sodium acetate trihydrate (pH 4.6) and 25 U of amyloglucosidase were added to each tube, and samples were incubated overnight at 70°C to complete starch hydrolysis to glucose. The next day, samples were incubated at 100°C for 5 min to inactivate the enzyme, then vortexed and centrifuged at 22,000 g. Glucose resulting from starch hydrolysis was quantified based on NADPH production, following the method of Bernt and Bergmeyer (1974). This assay involved adding 200 μL of a reaction buffer containing 50 mM Tris–HCl (pH 7.5), 2 M MgCl_2_, 50 mM NADP+ in Tris, and 0.4 M NaATP in Tris to each well of a Victor microplate. An initial spectrophotometric reading at 340 nm was taken to obtain basal NADPH levels for each sample. Each well then received 0.2 U of hexokinase and 0.5 U of glucose‐6‐phosphate dehydrogenase, both suspended in 100 μL of reaction buffer. The enzymatic reaction was conducted at 37°C for 20 min, followed by a final spectrophotometric reading to determine NADPH production. The glucose content was calculated by subtracting the initial from the final absorbance readings and comparing the result to a calibration curve with known glucose concentrations.

#### 2.3.5 Carbon Isotope δ^13^C Composition

Berry samples from watered, D2‐Moderate, and D2‐Severe treatments were collected and immediately conserved at −20°C until analysis. For each treatment, at least 500 g of berries were sampled, ensuring homogeneous collection from all plants within the respective treatment group. δ^13^C isotope composition analyses were performed by an officially accredited laboratory (WhiteLab, U‐Series Srl, Bologna, Italy; https://whitelab.it/it/labs/u‐series/) using the official UNI ENV 12140:1997 procedure. Briefly, about 30 g of frozen sample was collected, squeezed, and filtered to produce 25 mL of juice. Then, Ca(OH)_2_ was added until the pH reached 8.5. After a passage inside a thermostatic bath set at 80°C for 15 min, the samples were centrifuged for 10 min at 1070 g. The supernatant was picked up and acidified to pH 5 with H_2_SO_4_ 0.1 M. After an overnight cooling in the fridge, 1 μL of the supernatant was collected and dried in the oven (45°C for 45 min). Finally, stable isotope analysis was conducted on samples composed of purified grape sugars by using a High‐Temperature Conversion Elemental analyser (TC/EA) coupled with a Delta V Advantage Isotope Ratio Mass Spectrometer (Thermo Fisher Scientific GmbH). Each analysis was conducted on three replicates. The calculation of the carbon isotope ratio (δ^13^C) was δ^13^C_sample_ (‰) = (*R*_sample_/*R*_standard_ − 1) × 1000 (Farquhar and Richards 1984), where *R*_sample_/*R*_standard_ were referred to a Pee Dee Belemnite (PDB) standard.

### 2.4 Statistical Analysis

The experimental design was completely randomized. Statistical analysis was conducted using R software version 4.3.1. A one‐way ANOVA was performed to assess differences between treatments at each time point for the variables sap flow, Photosystem II efficiency (ΦPSII), and isotope composition, followed by Tukey's post hoc test when significant interactions were found.

For NSC, a two‐way ANOVA was conducted to evaluate the effects of both time and treatment, followed by Tukey's post hoc test when significant interactions were detected. Assumptions of normality and homoscedasticity were tested using the Shapiro–Wilk test and Levene's test, respectively.

For the other variables of chlorophyll fluorescence, gas exchange, and water potential which did not follow a normal distribution, a non‐parametric Kruskal–Wallis test was applied to compare differences between treatments. When significant results were found, the Dunn test with Bonferroni correction was used for pairwise comparisons. Differences were considered statistically significant at the *p* ≤ 0.05 level, except for isotope discrimination, where a more stringent threshold of *p* ≤ 0.001 was applied.

## 3 Results

### 3.1 Vine Water Status and Transpiration Dynamics

**FIGURE 3:**
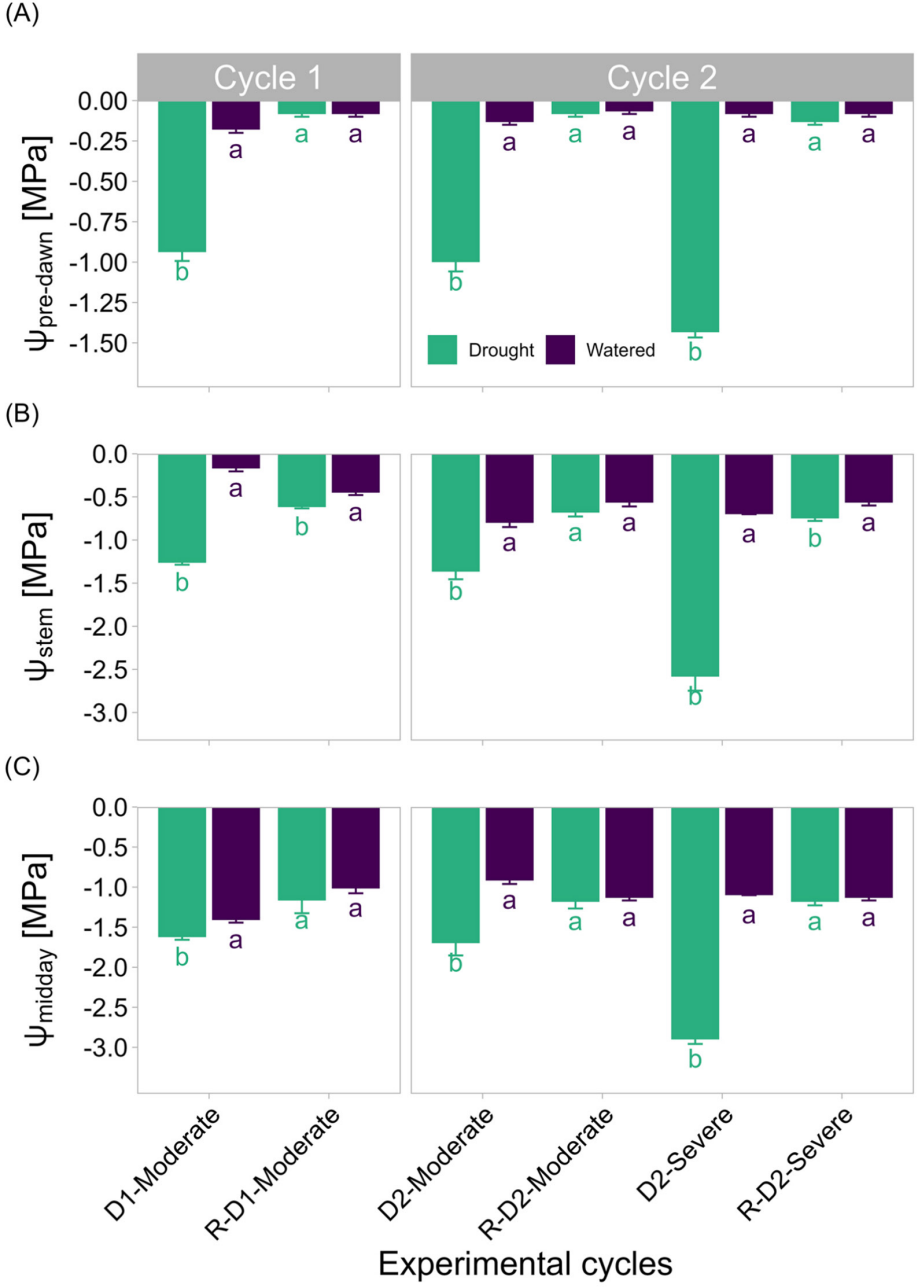
Leaf and stem water potential under watered and drought conditions across multiple drought‐rewatering cycles. Values are shown for Ψ_pre‐dawn_ (A), Ψ_stem_ (B), and Ψ_midday_ (C) under watered (purple) and drought (green) conditions. Measurements include the first moderate drought phase (Drought 1‐Moderate), rewatering (R‐D1) and subsequent second Moderate (D2‐Moderate) and Severe drought events (D2‐Severe), and their corresponding recovery phases (R‐D2‐Moderate and R‐D2‐Severe). Values represent the mean ± SE. Significant differences between watered and drought stressed groups at each time point are indicated by different lowercase letters according to Dunn test with Bonferroni correction (*p* ≤ 0.05; *n* = 6 leaves per treatment).

To monitor the plant water status, Ψ_pre‐dawn_ and Ψ_midday_ as well as Ψ_stem_ were measured (Figure 3). In watered plants, Ψ_pre‐dawn_ remained stable and close to 0 MPa throughout the measurement days (Figure 3A). In contrast, drought‐stressed plants exhibited Ψ_pre‐dawn_ values near −1 MPa during D1 and D2‐Moderate drought, which dropped to approximately −1.5 MPa during D2‐Severe drought, indicating that already during D1 and D2‐Moderate drought, plants were experiencing considerable water stress. During rewatering periods, according to Ψ_pre‐dawn_ values, drought‐stressed plants were comparable to the water status of the well‐watered ones (Figure 3). For Ψ_stem_, watered plants maintained steady values around −0.5 MPa (Figure 3B). In stressed plants, Ψ_stem_ was used as a reference parameter to drive drought imposition and reached values of about −1.3 MPa during D1 and D2‐Moderate drought, and around −2.5 MPa during D2‐Severe drought.

**FIGURE 4:**
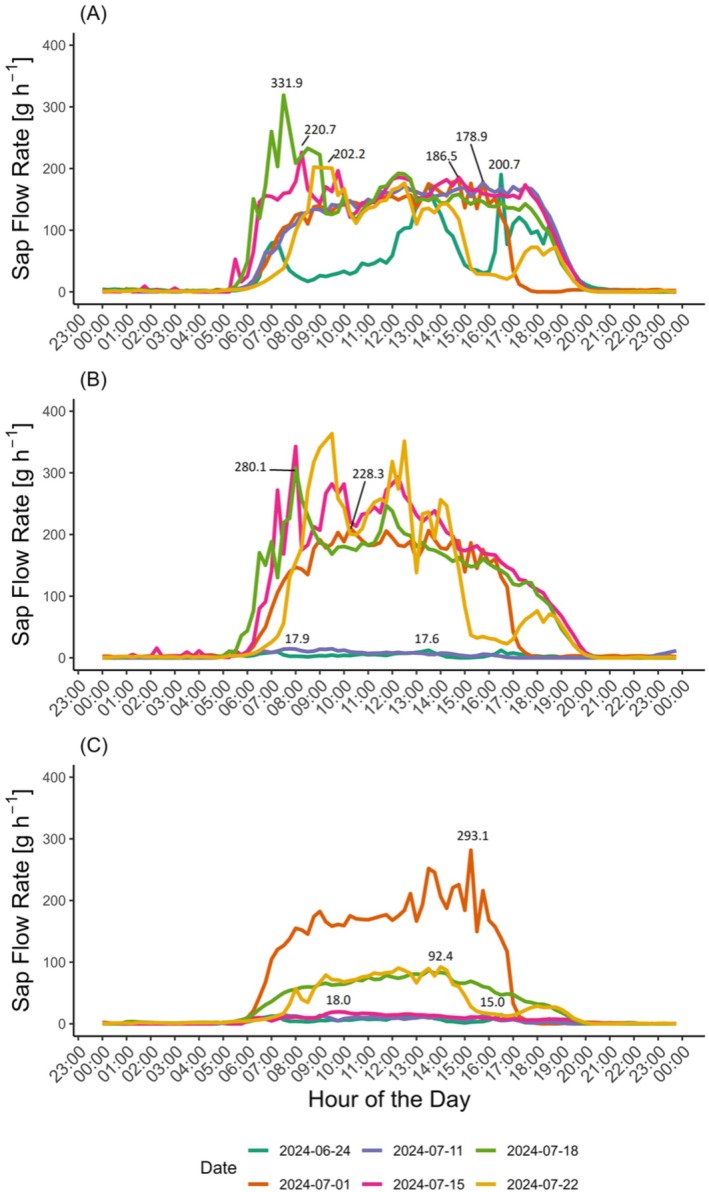
Daily pattern of relative sap flow rates throughout the experiment. Values show the relative sap flow rates throughout the day, averaged across three replicates per treatment. Each colored line represents the time course of 1 day: (A) watered plants, (B) second moderate (D2‐Moderate) and (C) severe drought events (D2‐Severe; *n* = 3 plants per treatment).

Under the drought conditions imposed, the measured sap flow rates showed a clear daily pattern across all the three treatments (watered, D2‐Moderate, and D2‐Severe drought). The rates began with a rapid increase just after sunrise (5:00–7:00 h), followed by consistently high and stable flow rates (between 150 and 300 g h^−1^) from approximately 8:00 h until around 17:00 h and afterwards reaching their peak; the rates sharply declined until 0 g h^−1^ (Figure 4 and Figure S1). As expected, watered plants consistently showed higher transpiration rates on measurement days as compared to D2‐Moderate and D2‐Severe ones, except for the 24 June, when cloudy weather and precipitation occurred (Figure 4A). For the D2‐Moderate (Figure 4B) and D2‐Severe (Figure 4C) treatments, the sap flow rates changed in accordance with the trends observed on each measurement day. Until water was available, the sap flow rate was high; conversely, upon water stress imposition, the sap flow rates dropped to nearly 0 g h^−1^. The numbers reported near to the lines indicate the maximum sap flow rate for the respective day of measurement.

### 3.2 Leaf Gas Exchange Parameters

**FIGURE 5:**
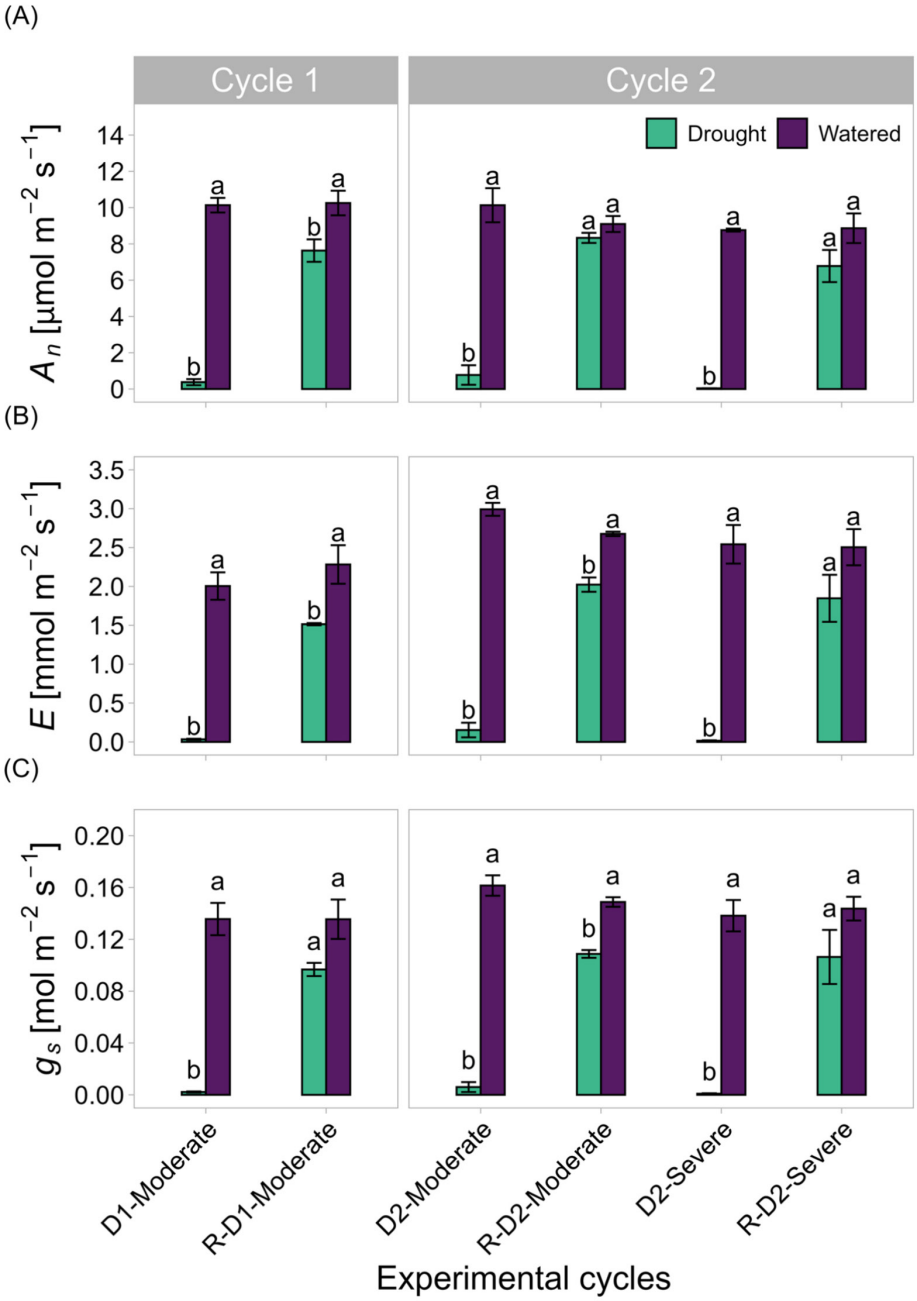
Gas exchange measurements in grapevine leaves under watered and drought conditions across multiple drought‐rewatering cycles. Parameters measured include net assimilation rate (*A*_*n*_, μmol CO_2_ m^−2^ s^−1^), transpiration rate (*E*, mmol H_2_O m^−2^ s^−1^), and stomatal conductance (*g*_*s*_, mol H_2_O m^−2^ s^−1^) on leaves under watered (purple) and drought (green) conditions. Measurements include the first moderate drought phase (Drought D1‐Moderate), rewatering (R‐D1) and subsequent second moderate (D2‐Moderate) and severe drought events (D2‐Severe), and their corresponding recovery phases (R‐D2‐Moderate and R‐D2‐Severe). Values represent the mean ± SE. Significant differences between watered and drought stressed groups at each time point are indicated by different lowercase letters according to Dunn test with Bonferroni correction (*p* ≤ 0.05; *n* = 3 leaves per treatment).

Plants exhibit coordinated responses to fluctuations in water availability, adjusting *A*_*n*_ (net assimilation), *E* (transpiration rate), and *g*_*s*_ (stomatal conductance) during cycles of drought and rewatering (Figure 5). Watered plants maintained steady *A*_*n*_ rates between 9 and 11 μmol CO_2_ m^−2^ s^−1^ throughout the experiment. Plants under drought exhibited a marked reduction in *A*_*n*_ during drought, dropping to approximately 0–2 μmol CO_2_ m^−2^ s^−1^ at peak stresses. Notably, plants under drought demonstrated an ability to recover between stress periods, with *A*_*n*_ values returning to near watered levels upon rewatering. Although R‐D2‐Severe plants under drought exhibited slightly lower recovery in *A*_*n*_, with values around 6 μmol CO_2_ m^−2^ s^−1^ compared to approximately 8 μmol CO_2_ m^−2^ s^−1^ in well‐watered plants. This difference was not statistically significant (Figure 5A).

During rewatering, stomatal reopening allows for the restoration of gas exchange and water flow, normalizing *E* and *A*_*n*_ levels (Figure 5B). In watered plants, *g*_*s*_ remained stable, ranging from 0.12 to 0.15 mol H_2_O m^−2^ s^−1^. By contrast, plants under drought exhibited near‐zero *g*_*s*_ during drought periods, indicating that Sauvignon blanc experienced drought conditions leading to full stomatal closure. In rewatering phases, *g*_*s*_ returned to baseline levels (Figure 5C and Figure S2).

Transpiration rate (*E*), representing water lost through stomata, increased for watered plants at D2‐Moderate, peaking at around 3 mmol H_2_O m^−2^ s^−1^ before stabilizing at 2.5 mmol H_2_O m^−2^ s^−1^ (Figure 5B). Conversely, plants under drought exhibited a sharp decrease in *E* during water deprivation, with values of transpiration approaching zero. In rewatering phases, vines under drought regained physiological activity, though their *E* rates remained significantly below their watered levels, except after the D2‐Severe drought.

### 3.3 Chlorophyll Fluorescence

**FIGURE 6:**
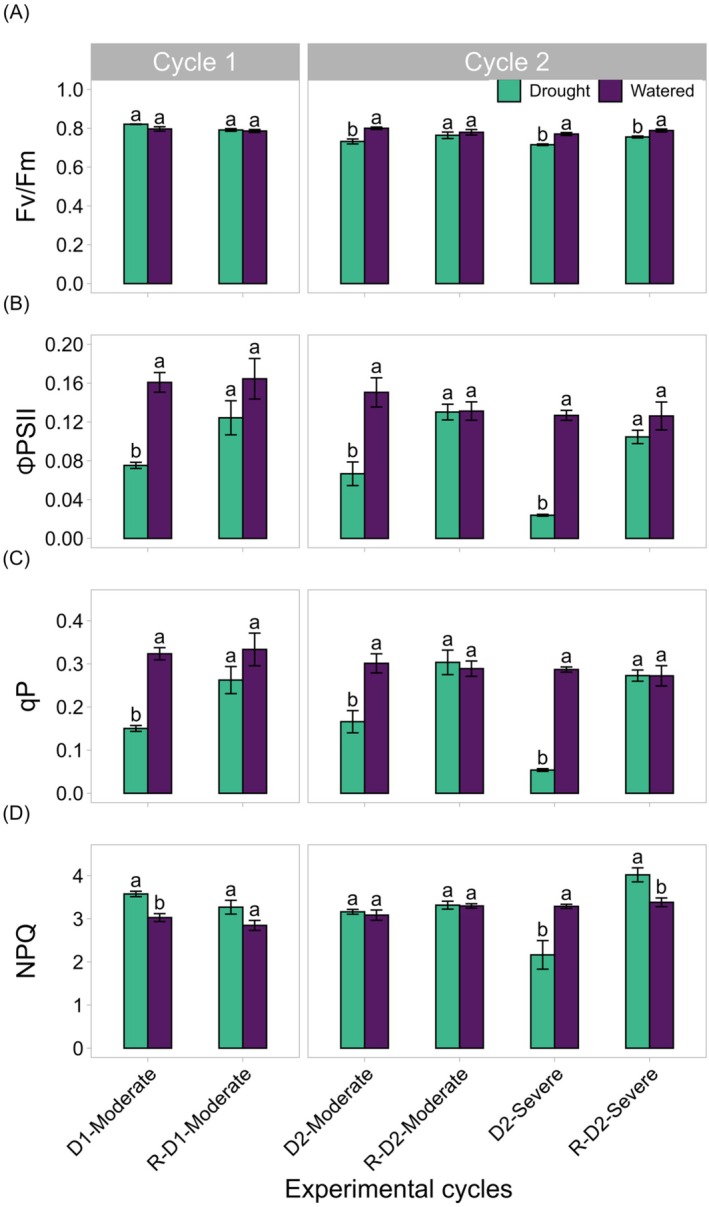
Chlorophyll fluorescence parameters under watered and drought conditions. The figure shows maximum efficiency of the Photosystem II (Fv/Fm), steady‐state efficiency of the PSII (ΦPSII), photochemical quenching (qP) and non‐photochemical quenching (NPQ) on leaves under watered (purple) and drought (green) conditions. Measurements include the first moderate drought phase (Drought D1‐Moderate), rewatering (R‐D1) and subsequent second Moderate (D2‐Moderate) and Severe drought events (D2‐Severe), and their corresponding recovery phases (R‐D2‐Moderate and R‐D2‐Severe). Values represent the mean ± SE. Significant differences between watered and drought stressed groups at each time point are indicated by different lowercase letters according to Dunn test with Bonferroni correction (*p* ≤ 0.05), except for ΦPSII where Tukey's test was applied (*p* ≤ 0.05; *n* = 3 leaves per treatment).

Four key chlorophyll fluorescence parameters were monitored throughout the drought–rewatering cycles. In well‐watered plants, the Fv/Fm ratio remained stable over the entire experimental period, consistently around 0.80, indicating no significant stress. In contrast, drought‐stressed plants exhibited marked reductions in Fv/Fm during the drought phases, with values ranging between 0.71 and 0.75 in drought plants in comparison to 0.78 and 0.80 in watered plants (Figure 6A).

ΦPSII and photochemical quenching (qP) exhibited similar trends across treatments. During drought cycles, values in drought‐stressed plants were consistently lower than those in well‐watered plants. Specifically, qP ranged from 0.05 to 0.17 in drought‐stressed plants, compared to values between 0.28 and 0.32 in well‐watered controls. Similarly, ΦPSII values ranged from 0.02 to 0.06 and from 0.12 to 0.16 in drought‐stressed and well‐watered plants, respectively (Figure 6B,C).

The effectiveness of non‐photochemical energy dissipation systems was measured by the non‐photochemical quenching (NPQ) index, which is primarily associated with the efficacy of the xanthophyll cycle. During the D2‐Moderate drought, the NPQ values in plants under drought were similar to those of the watered ones (Figure 6D). Low NPQ values (2.16) were measured in drought‐stressed plants during D2‐Severe drought, but during the rewatering (R‐D2‐Severe) the same plants showed higher values than watered plants (4.00 vs. 3.38; Figure 6D).

**FIGURE 7:**
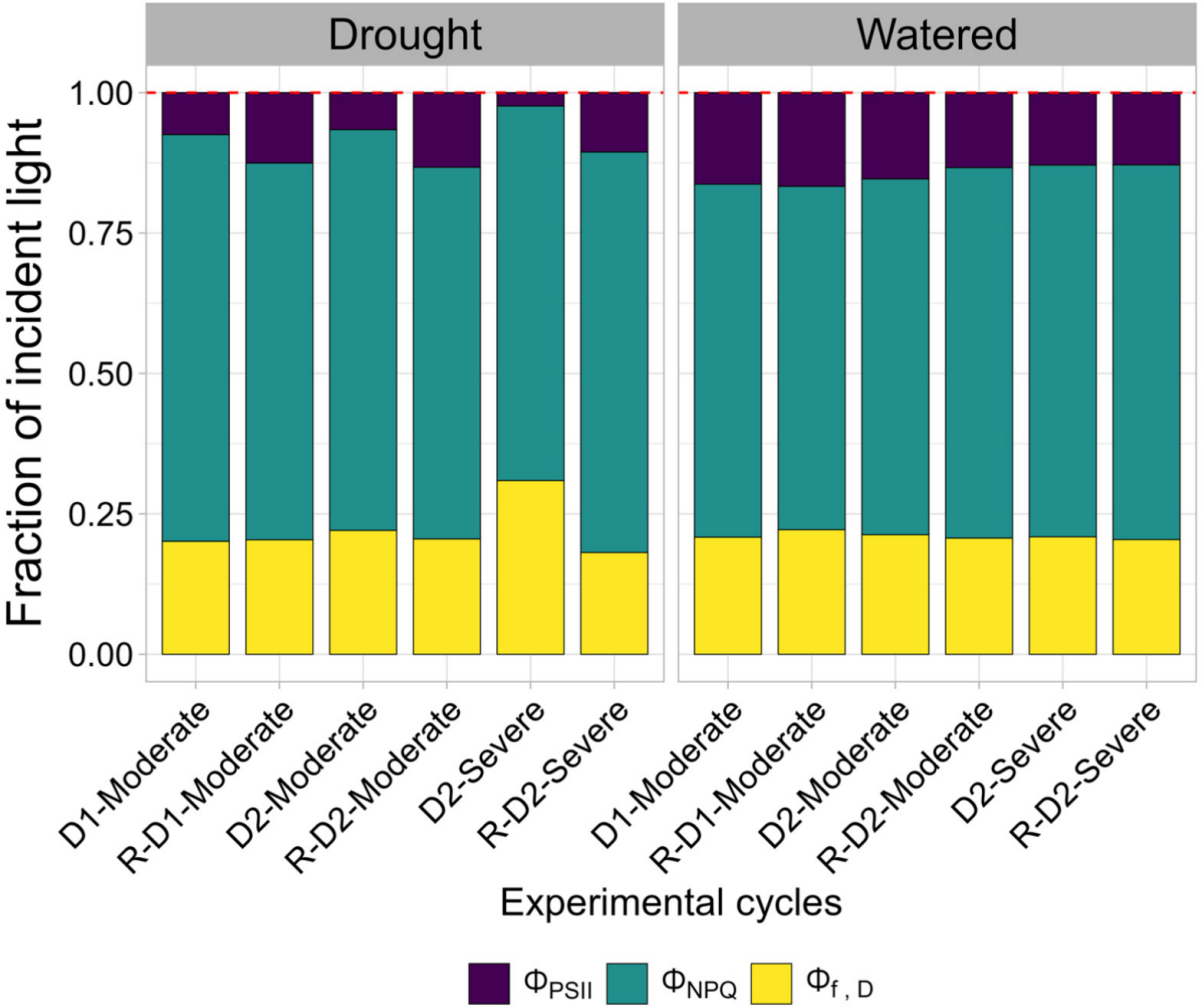
Estimated partitioning of absorbed irradiance in grapevine leaves under multiple drought‐rewatering cycles. Values show the fraction of absorbed irradiance consumed via photochemistry (ΦPSII), ΔpH‐ and xanthophyll‐regulated thermal dissipation (Φ_NPQ_), and the sum of fluorescence and constitutive thermal dissipation, (Φ_f,D_). Measurements include the first moderate drought phase (D1‐Moderate), rewatering (R‐D1) and subsequent second moderate (D2‐Moderate) and severe drought events (D2‐Severe), and their corresponding recovery phases (R‐D2‐Moderate and R‐D2‐Severe; *n* = 3 leaves per treatment).

Additionally, the partitioning of excitation energy among photochemistry, fluorescence, and thermal dissipation was evaluated (Figure 7; Hendrickson et al. 2004; Losciale et al. 2011). The figure illustrates how incident light was distributed under drought and well‐watered conditions, revealing a reduction in the proportion of absorbed irradiance used for photochemistry (Φ_PSII_), alongside an increase in regulated thermal dissipation (Φ_NPQ_), as well as in the sum of fluorescence and constitutive thermal dissipation (Φ_f,D_) in drought‐stressed plants.

### 3.4 Carbon Isotope Composition

The evaluation of carbon isotope ratios provides a valuable parameter for assessing the water status experienced by plants throughout their development cycle. Our results show significant differences between all three treatments (Table 1), effectively distinguishing both from the watered and between the two drought levels. Berries from the watered treatment showed the most negative δ^13^C value (−26.31). Under moderate drought (D2‐Moderate), δ^13^C values were significantly (*p* < 0.001) less negative (−25.87), followed by D2‐Severe, which was the most enriched in δ^13^C (−25.01). These results indicate a significant enrichment in ^13^C in berries under increasing drought stress.

**TABLE 1:** Isotopic fraction (δ^13^C) of berries collected from watered and drought conditions.

Treatment	δ^13^C vs. VPDB	*p* < 0.001
Watered	−26.31 ± 0.021	a
D2‐Moderate	−25.87 ± 0.070	b
D2‐Severe	−25.01 ± 0.081	c

*Note:* Results are expressed as ‰ deviation from the international VPDB standard. Significant differences between groups are indicated by lowercase letters according to Tukey's test (*p* ≤ 0.001).

### 3.5 Non‐structural Carbohydrates

**FIGURE 8:**
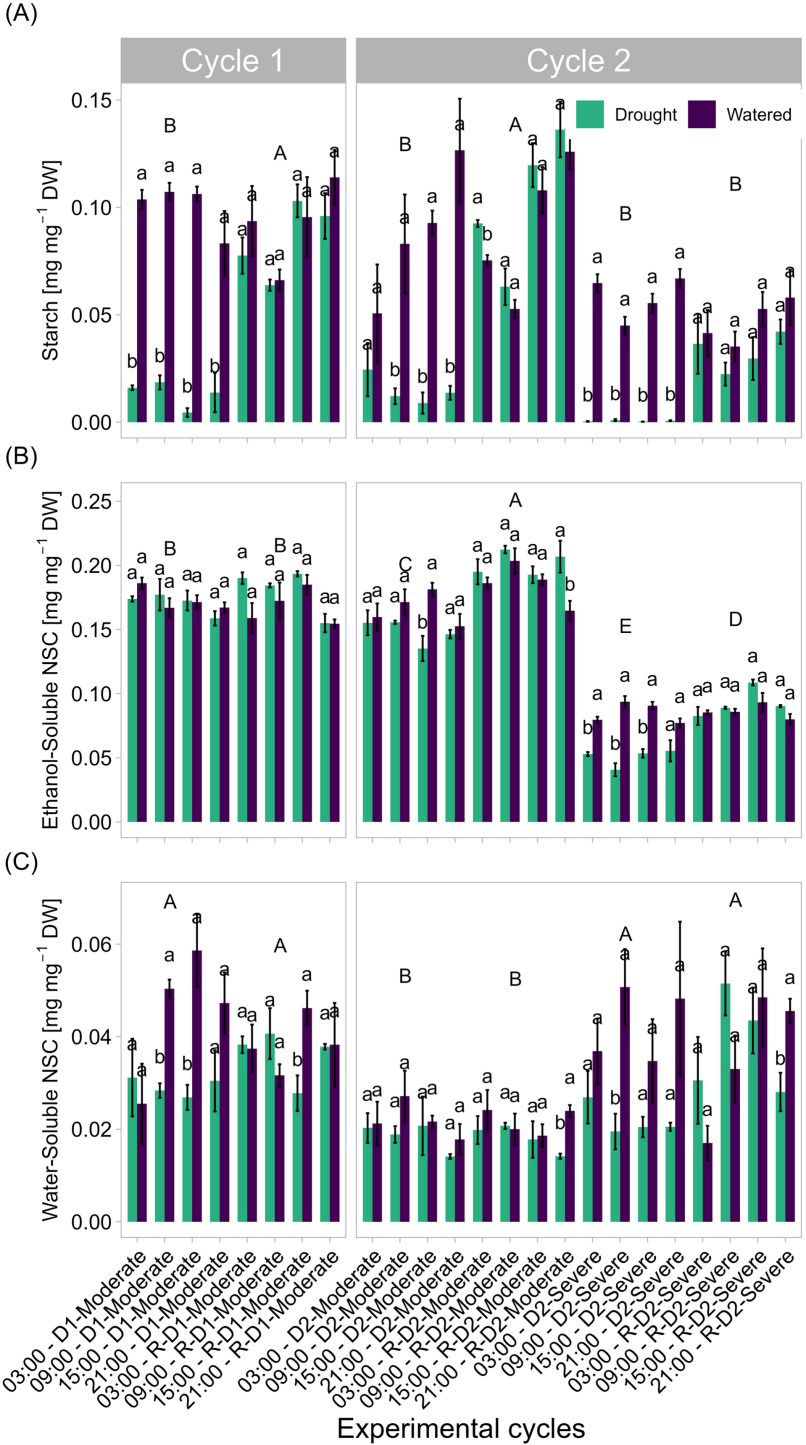
Nonstructural carbohydrate (NSC) content daily dynamics in watered and drought‐stressed leaves. Starch (A) ethanol‐soluble (B) and water‐soluble (C) results are expressed in mg per mg of dry weight. Each bar represents the NSC content of samples collected at different phases (D1, D2‐Moderate, D2‐Severe and their respective rewatering phases) and at different times of the day (3:00, 9:00, 15:00, and 21:00 h). Values represent the mean ± SE. Significant differences between watered and drought groups at each time point are indicated by different lowercase letters, whereas differences between cycles are indicated by capital letters according to Tukey's test (starch: cycle *p* < 0.001, treatment *p* < 0.001, cycle × treatment *p* < 0.001; ethanol‐soluble: cycle *p* < 0.001, treatment *p* < 0.05, cycle × treatment *p* < 0.01; water‐soluble: cycle *p* < 0.001, treatment *p* < 0.001, cycle × treatment *p* < 0.05).

The NSCs analysis was performed during the D1, D2‐Moderate, and D2‐Severe drought cycles and their respective rewatering phases. The quantification of NSCs in leaves discriminated between soluble forms (simple, ethanol‐soluble sugars, and glucans, water‐soluble) and starch. As shown in Figure 8A, starch was strongly reduced by both moderate drought treatment (D1 and D2‐Moderate), independently from the hour of the day, and completely depleted in D2‐Severe. During the rewatering period, starch content recovered to levels comparable to or even exceeding those of well‐watered plants, as observed at 3:00 h in the R‐D2‐Moderate treatment (Figure 8A). As expected, ANOVA results revealed a significant interaction between drought treatment and cycle, indicating that the effect of drought on starch content varied depending on the day cycle. No significant differences were observed between drought and well‐watered treatments during D1, R‐D1, D2‐Moderate, R‐D2‐Moderate, and R‐D2‐Severe, except at 15:00 h for D2‐Moderate and at 21:00 h for R‐D2‐Moderate in ethanol‐soluble carbohydrates (Figure 8B). In contrast, under D2‐Severe conditions, significant differences between drought and watered treatments were observed at all four different time points analyzed. Water‐soluble NSCs (i.e., glucans and branched glucans) decreased significantly under drought at several time points, as shown by the lowercase letters (Figure 8C). Even during the recovery phases (R‐D1, R‐D2‐Moderate, and R‐D2‐Severe), drought‐stressed plants struggle to restore their levels, which remain lower than those of the watered ones.

## 4 Discussion

The results partially support the hypothesis that repeated exposure to water deficit negatively affects plant recovery capacity. The first and second moderate drought did not substantially alter leaf gas exchange or chlorophyll fluorescence, as initially expected, although they did affect NSC and carbon isotope composition. In contrast, the second severe drought cycle led to more extensive physiological changes, also altering chlorophyll fluorescence. However, it remains unclear whether these effects were due to the cumulative stress or simply the severity of the second drought event. For this reason, we did not interpret the second stress as a cumulative effect, but rather as an independent severe event. Here, we discussed how grapevines responded to this severe stress—with water potentials lower than any previously reported in the literature—and how, despite losing 30%–50% of their leaves, the plants still showed a remarkable capacity to recover from stress. In particular, we highlighted the plant's distinct responses in terms of chlorophyll fluorescence and diurnal patterns of NSC accumulation.

Upon rewatering, plants subjected to the second D2‐Severe drought (R‐D2‐Severe) were unable to recover the water potential as efficiently as those exposed to the D1 (R‐D1) and D2‐Moderate (R‐D2‐Moderate) treatments. These results suggest that the D2‐Severe drought may have caused deeper physiological damage, such as loss of hydraulic conductivity, depletion of carbon reserves, and diminished cambial activity, all of which likely hindered the plants' ability to recover effectively after rewatering (Hochberg, Bonel, et al. 2017; Hochberg, Windt, et al. 2017; Sorek et al. 2021). It is worth noting that these stress levels were achievable due to the use of a highly porous substrate in 30 L pots. Interestingly, a faster decline in soil moisture was observed in the second drought cycle (D2‐Moderate) compared to the first (D1‐Moderate), as it took only 4 days to reach target stress levels in D2 versus 6 days in D1. This difference may be attributed mainly to the lower water retention capacity of the substrate and to higher daytime temperatures during the D2 phase, which likely contributed to accelerated soil drying. The leaf water potential measured at midday (Figure 3C) corresponds to the peak of water constraints for the plant as transpiration losses reach their highest rates and are not paired by water absorption by the roots; this is occurring also under nonlimiting water availability (Deloire et al. 2020; Tardieu and Simonneau 1998). Indeed, at midday, watered plants experienced such a slight water stress condition with Ψ_midday_ values of about −1 MPa. In drought‐stressed plants, on the contrary, Ψ_midday_ values dropped to approximately −1.5 MPa under D1 and D2‐Moderate drought, whereas D2‐Severe drought triggered a much stronger response, with Ψ_midday_ peaking at −3 MPa (Figure 3C). Notably, plants in the D2‐Severe treatment experienced leaf shedding of about 30%–50% of their leaves (Figure S3), a condition that can lead to very low Ψ_midday_ has also been reported in the literature (Charrier et al. 2018; Sorek et al. 2023). This response is often associated with hydraulic dysfunction under drought stress. In particular, recent findings suggest that leaf embolism is a more reliable indicator of drought‐related damage than stem embolism (Brodribb et al. 2021) and may be more directly linked to leaf shedding (Hochberg, Windt, et al. 2017), thereby highlighting the importance of monitoring leaf‐level hydraulic failure in assessing plant responses to severe water deficit.

Relevant differences were observed also in the sap flow rates. Although plants subjected to D2‐Moderate drought were able to recover after rewatering (R‐D2‐Moderate), those exposed to D2‐Severe drought (R‐D2‐Severe) showed a significantly impaired recovery, with a more pronounced reduction in sap flow rates compared to both the well‐watered plants and those from the R‐D1 treatment. Data analysis suggests that while D2‐Moderate plants showed higher maximum sap flow rates after water stress imposition, indicating a significant recovery, D2‐Severe plants exhibited consistently lower maximum rates during the rewatering period. Even after rewatering, sap flow rates in D2‐Severe plants remained lower compared to D2‐Moderate and watered plants, pointing to a less pronounced recovery response. These findings indicate that D2‐Severe plants likely encountered more severe constraints in re‐establishing sap flow following stress, compared to the more effective recovery seen in D2‐Moderate plants. The results are in agreement with those reported by Benyahia et al. (2023), which showed that daily sap flow patterns were highly affected by the intensity of the water stress.

Different from our original hypothesis, leaf gas exchange parameters were restored in both moderate and severe drought conditions. Studies suggest that in severe stress conditions, stomatal closure limits photosynthesis (Flexas et al. 2004). However, in our case, the lack of a significant difference in recovery suggests that no major metabolic impairment occurred. Stomatal closure is an early plant response to water deficit, aimed at minimizing water loss through reduced transpiration, but it also limits CO_2_ uptake (Dayer et al. 2019). Pou et al. (2008) demonstrated that potted vines of the *Vitis* hybrid Richter‐110 ( *Vitis berlandieri* × *Vitis rupestris* ) exposed to comparable levels of water stress (midday Ψ_stem_ ranging from −1.4 to −1.5 MPa) required several days, and in some cases up to 2 weeks, for stomatal conductance to fully recover. Charrier et al. (2018) found that the duration of the rewatering period for vines is longer as the severity of the drought increases. Additionally, Tombesi et al. (2018) emphasized that potted vines of cv. Sangiovese and Montepulciano subjected to drought exhibited altered stomatal regulation even after being relieved from stress (following rewatering). Nevertheless, it should be acknowledged that in D2‐Severe plants, recovery occurred in a reduced canopy, as shedding during the stress period left only about half of the original leaf area. Thus, although *gs* measured at the leaf level appeared to recover, whole‐plant transpiration likely remained below pre‐stress values (see Figure 4 and Table 2).

**TABLE 2:** Summary of the maximum sap flow rates on different dates showcasing the variations in their responses to water stress and recovery.

Date	Drought cycle 1		Drought cycle 2			
	24 June	1 July	11 July	18 July	15 July	22 July
Treatment	Drought moderate 1 (D1)	Rewatering (R‐D1)	Drought moderate 2 (D2‐Moderate)	Rewatering (R‐D2‐Moderate)	Drought severe 2 (D2‐Severe)	Rewatering (R‐D2‐Severe)
Watered	200.7 ± 5.5 a	186.5 ± 3.8 c	178.9 ± 1.1 a	331.9 ± 7.2 a	220.7 ± 8.1 a	202.2 ± 2.0 a
D2‐Moderate	17.6 ± 0.7 b	228.3 ± 10.8 b	17.9 ± 1.8 b	280.1 ± 35.3 a		
D2‐Severe	15.0 ± 2.6 b	293.1 ± 5.9 a			18.0 ± 3.2 b	92.4 ± 4.4 b

*Note:* The “treatment” and “date” rows indicate the specific experimental cycle and the exact dates on which physiological measures were taken, respectively. The watered row represents the sap flow rates measured under well‐watered conditions throughout the experiment (watered plants). The D2‐Moderate and the D2‐Severe rows report the maximum sap flow rates recorded in plants that underwent moderate and severe drought, respectively. Each column reflects the sap flow data for the respective plant treatment on the specific measurement day, allowing for a comparison of how different stress durations affected the plants' physiological responses. Values represent the mean ± SE. Significant differences between control and drought groups at each time point are indicated by different lowercase letters according to Tukey's test (*p* ≤ 0.05).

Overall, drought‐stressed grapevines displayed acclimative water‐conserving strategies such as stomatal closure that resulted in significant reductions in gas exchange. These physiological adjustments and others—such as improved water retention and a partial recovery of photosynthetic activity upon rewatering—suggest that the plants may have developed mechanisms to minimize water loss and maintain a basal level of photosynthesis even under fluctuating water availability (Tombesi et al. 2018). The observed recovery pattern indicates that the initial drought (D1) did not cause permanent damage to the stomatal or photosynthetic apparatus but rather triggered an acclimative response, suggesting a form of “drought memory” that enhances plant resilience under recurring drought conditions (Tombesi et al. 2018). On the other hand, although the plants did not exhibit any impairment in leaf gas exchange following moderate or severe drought, changes in chlorophyll fluorescence were observed. Chlorophyll fluorescence analyses were measured as an indirect method used to assess the efficiency of photosynthesis (Galat Giorgi et al. 2019; Murchie and Lawson 2013; Schreiber et al. 1995). The Fv/Fm ratio, which represents the maximum efficiency of Photosystem II (ΦPSII) and is the most commonly used index for measuring stress intensity in plant leaves (Murchie and Lawson 2013), remained stable in watered plants throughout the experimental period, consistently showing values around 0.80 that are considered as normal values in grapevines (Bernardo et al. 2022; Ju et al. 2018; Palliotti et al. 2015). In contrast, plants under drought showed significant reductions in Fv/Fm ratios during the drought phases. A similar decrease was reported by Benyahia et al. (2023), where the Fv/Fm index also declined significantly during both drought cycles, although the values reported in their study were lower than those measured in the present work. Nonetheless, during the rewatering phases, plants under drought were able to partially restore their Fv/Fm values and significant differences were found at the second cycle among the moderate and severe drought groups. Notably, in R‐D1 and R‐D2‐Moderate, the recovery was complete, and vines regained the physiological status of their watered plants. However, after experiencing D2‐Severe drought, the recovery did not fully restore the Fv/Fm values to normal levels. The incomplete recovery of Fv/Fm values following D2‐Severe drought could be attributed to the more severe nature of the drought. Prolonged water stress can lead to photoinhibition or the accumulation of reactive oxygen species, which may impair the repair mechanisms of PSII or cause irreversible damage to its proteins and pigments (Gururani et al. 2015).

ΦPSII, which reflects the instantaneous efficiency of Photosystem II under light‐adapted conditions, is directly related to the amount of energy directed toward photosynthesis under field conditions (Kramer et al. 2004). The reduction of ΦPSII during drought correlates with a decrease in the phototransduction capacity of plants. Water stress disrupts the normal functioning of electron transport by limiting the availability of water needed for electron donation, thereby impairing the energy flow required for photosynthesis. This disruption is evident in the qP parameter, which reflects the proportion of open PSII reaction centers available for light absorption during photosynthesis. A reduction in the number of open reaction centers decreases light energy efficiency, leading to an overall decline in photosynthetic performance during the drought phase. As qP decreases, this reduction is further corroborated by the significant decreases observed in Fv/Fm and ΦPSII parameters. These reductions were particularly pronounced during all drought cycles, with the most substantial decline occurring during the D2‐Severe drought, highlighting the plant's diminished capacity to maintain open ΦPSII reaction centers under severe stress conditions. The effectiveness of non‐photochemical energy dissipation systems was measured by the NPQ index, which is primarily associated with the efficacy of the xanthophyll cycle. Under drought conditions, NPQ typically increases as an adaptive response to prevent photodamage by dissipating excess energy that cannot be utilized in photosynthesis due to drought‐induced limitations (Schreiber et al. 2008). The D2‐Moderate drought represents a relatively brief drought period, where short‐term stress likely leads to a temporary reduction in photosynthetic efficiency without severely affecting Photosystem II functionality. It is also possible that prior drought and rewatering cycles “primed” the plants, enhancing Photosystem II repair mechanisms, which minimized the photoprotective response (NPQ) to levels similar to the watered plants. The contrasting NPQ values observed between D2‐Severe (low NPQ) and its recovery, R‐D2‐Severe (high NPQ), suggest a shift in the plant's protective strategy due to prolonged stress exposure. Low NPQ values measured during D2‐Severe drought likely indicate a suppressed or depleted photoprotective response under sustained stress, possibly due to a reduction of ΦPSII, confirmed also by the fraction of incident light (Figure 7). In contrast, the higher NPQ values registered for plants under drought as compared to the watered ones during the rewatering (R‐D2‐Severe) reflect the reactivation of the photoprotective response as the plants adjusted to the restored water availability. As photosynthesis begins to recover, the plant may increase NPQ before greater light absorption, even if photosynthetic efficiency remains temporarily low (Murchie and Ruban 2020). Additionally, plants may exhibit “stress memory” where responses to prolonged drought, such as hormonal signaling (e.g., ABA), persist after rewatering (Tombesi et al. 2018). This could result in a sustained higher NPQ, acting as a protective buffer in case drought conditions return. The absence of Fv/Fm reduction at D1 aligns with what was observed by Palliotti et al. (2015). Authors noted in Montepulciano (isohydric) and Sangiovese (anisohydric) grapevines that the maximum efficiency of the photosystem was unaffected by water stress before veraison. This may be due to the greater plantcapability until veraison to allocate resources to the protection of the photosystem, as highlighted in our NPQ dynamics. Eventually, our results highlight the plant's inability to recover the Fv/Fm after severe drought and an interesting strategy to anticipate the heat dissipation, increasing NPQ, before greater light absorption.

NSC dynamics evidenced grapevines strategies to be prepared for successive stress. Our results confirmed that leaf NSC is mostly represented by soluble sugars (Perry et al. 2025; Tombesi et al. 2021; Vuerich et al. 2021). Starch, a transient storage form of NSCs in leaves, accumulates in the chloroplasts (Martínez‐Vilalta et al. 2016) and plays a key role in short‐term carbon management rather than in long‐term storage. Interestingly, in this case, starch is not reduced during the night as expected (Graf and Smith 2011; Stitt and Zeeman 2012), a phenomenon that was observed by Perry et al. (2022) in grapevine, where at 04:30 h the starch level was slower than at 15:00 h. In contrast, Tombesi et al. (2021) observed a complete starch depletion in the leaves of cv. Barbera during drought, but in this case, leaves were collected only at midday. Perry et al. (2025) observed a similar leaf pattern of NSC distribution during severe drought, suggesting a potential change from source to sink in leaf. However, our experiment demonstrated that it is not dependent on the day–night cycle and determined an accumulation of starch degradation product (i.e., both water and ethanol soluble NSC).

Notably, our study represents a novel approach by analyzing NSC dynamics over the day, including nighttime measurements. A striking finding was the high starch levels measured in grapevine leaves even at 3:00 h solar time, suggesting that under optimal water and light conditions, plants may experience a reduced need for osmotic regulation and/or can be due to specific characteristics of the species and/or phenological phase. Indeed, similar results were reported by Wingler and Henriques (2022). In their review, the authors discussed that under reduced growth conditions and limited sink demand (e.g., low temperatures or nitrogen deficiency), there is an accumulation of sucrose, trehalose‐6‐phosphate (Tre6P), and starch, suggesting that lower sink demand can lead to reduced nocturnal mobilization of NSCs. In Arabidopsis, dos Anjos et al. (2018) reported that under sink‐limiting conditions, with high starch rates of accumulation during the day, sucrose and Tre6P accumulated, and Tre6P inhibited starch breakdown, resulting in incomplete starch mobilization. Also, Resco De Dios and Gessler (2021) showed how the simultaneous limitation of sources and sinks affects the response of stored NSCs to drought in trees. A reduced sink demand can lead to lower nocturnal mobilization of NSCs, potentially altering carbohydrate dynamics and impacting the tree's ability to cope with water stress. Our hypothesis is that during the night, the absence of xylem transport may reduce the redistribution of solutes typically facilitated by water movement through the xylem, thereby limiting the availability of resources required for active physiological processes. Additionally, the thermodynamic constraints of the plant may prioritize nutrient allocation to the fruit rather than to the root, resulting in a decrease in the nocturnal offloading of metabolites. This altered distribution of resources could hinder the normal mobilization of NSCs, as their transport is closely tied to the plant's overall metabolic rhythms and water balance, which are disrupted in the absence of xylem flow. The dynamic tracking of NSCs across the day, including nighttime assessments, provides critical new insights into carbohydrate regulation under varying water availability.

Soluble NSC fraction is known to have smaller fluctuations than starch (Martínez‐Vilalta et al. 2016), reflecting the essential roles of soluble NSCs in cellular function: osmoregulation and energy supply for respiration, which maintain their levels above a critical threshold (Cho et al. 2022). These functions are sustained essentially by sucrose, fructose, and glucose (Ren et al. 2022), which account for most of the total sugars.

Interestingly, during the R‐D2‐Moderate recovery phase, stressed plants exhibited higher ethanol soluble NSC levels than controls, particularly with a pronounced end‐of‐day increase (21:00 h) (Figure 8B). This result can be explained by considering the role that NSCs play in osmotic adjustment in plants experiencing drought (Nawaz et al. 2025; Tomasella et al. 2020) and plant recovery (Pagliarani et al. 2019; Secchi and Zwieniecki 2016), recently confirmed also in grapevine (Vuerich et al. 2023). However, our findings are not completely in agreement with previous studies in which sugars were measured in petioles (Falchi et al. 2020; Vuerich et al. 2021), highlighting the relevance of collecting samples on a day‐cycle. The R‐D2‐Moderate phase was statistically distinct from the other ones, suggesting a priming effect, whereby plants, having already experienced an initial drought stress (D1), shifted their physiological response. One possible hypothesis is that instead of prioritizing growth, they accumulated greater soluble NSC reserves, likely as a strategy to enhance resilience to future stress events (McDowell 2011; Muller et al. 2011; Rehschuh et al. 2022), but further studies are necessary. This result supports the hypothesis that repeated drought cycles can induce a physiological “drought‐memory” effect, modifying carbon allocation patterns and reinforcing plant tolerance mechanisms (Kambona et al. 2023; Sadhukhan et al. 2022).

In contrast, water‐soluble NSCs (i.e., glucans and branched glucans) decrease significantly under stress at several time points. Even during the recovery phases (R‐D1, R‐D2‐Moderate, and R‐D2‐Severe), stressed plants struggle to restore their levels, which remain lower than those of the well‐watered plants, probably as a consequence of the decrease in starch. On the other hand, as suggested by Vuerich et al. (2023) working on grapevine stems, maltodextrins accumulation or reduction during recovery from drought depends on the hydraulic strategy of the cultivar (isohydric or anisohydric) and can be related to a different role in drought response. The more pronounced decline in the second stress cycle (D2‐Severe) highlights that severe drought has a deep impact, mirroring the trends observed for starch. The reduction may result from starch reduction and increased translocation to the vascular system, with levels recovering during subsequent rehydration. Starch constitutes the majority of NSCs and can be readily converted into soluble sugars when needed (MacNeill et al. 2017).

ANOVA analysis revealed a significant interaction between stress treatment and cycle, indicating that the effects of drought on ethanol‐soluble NSC and water‐soluble NSC were cycle‐dependent, with stress responses varying across different phases (Figure 8). This interaction showed that the impact of water stress on NSCs in the leaf was not uniform across the experimental cycles but varied depending on whether the plants were experiencing their first or second round of stress and recovery. Considering the metabolic fate of carbon assimilated by photosynthesis (Smith et al. 2005), our results suggest an interplay among different pools (i.e., hexoses, sucrose, starch, and glucans) that depends not only on water stress but also on its duration.

The carbon isotope composition was assessed as an integrative indicator of the plants' water stress status. Notably, the difference between D2‐Moderate and D2‐Severe drought plants is particularly striking. The δ^13^C values are reported relative to the international Vienna Pee Dee Belemnite (VPDB) standard, which serves as the reference for stable carbon isotope measurements. As demonstrated by Santesteban et al. (2012), water treatments applied just before harvest generally have a limited impact on isotopic fraction (δ^13^C). The significance of our findings may stem from the fact that our plants underwent water stress around veraison, a phenological stage identified by Taskos et al. (2020) as the period when δ^13^C is most sensitive to water deficits. In field conditions, veraison typically occurs 4 to 6 weeks before harvest (Santesteban et al. 2012), whereas in our experiment, this interval was shortened to about 2 weeks due to the accelerated phenology associated with plastic‐film tunnel cultivation. Consequently, the timing of the drought may have been too late to induce more pronounced effects on δ^13^C. Our results are consistent with those reported by Van Leeuwen et al. (2009), who provided a comprehensive assessment detailing carbon isotope variations occurring under different drought conditions.

## 5 Conclusions

Based on the data presented, we can conclude that the D2‐Severe drought cycle significantly impacted grapevine physiology, revealing both adaptive mechanisms and potential vulnerabilities. The observed changes in fluorescence parameters (Fv/Fm, ΦPSII, and NPQ) indicate that the plants experienced significant photosynthetic adjustments during repeated stress cycles. The incomplete recovery of Fv/Fm parameters together with the reduction of ΦPSII after the D2‐Severe suggests that severe water deficit may cause lasting damage to the photosynthetic apparatus, potentially limiting the plant's ability to fully recover under severe, extended drought conditions. On the other hand, the high NPQ, as a plant strategy to dissipate heat before greater light absorption, is a novel insight for grapevines' resilience. The NSC measurements reveal intricate carbohydrate dynamics in response to moderate and severe droughts. The higher ethanol‐soluble NSC levels observed during the R‐D2‐Moderate suggest a potential priming effect, where plants modify their carbon allocation strategies to enhance their resilience against future stress events.

Overall, these findings highlight grapevine capacity for physiological adaptations to moderate and severe drought events but also underscore the vulnerability under severe drought. This understanding of stress acclimation and adaptive responses is crucial for developing strategies to manage grapevines under increasingly variable climate conditions.

## Author Contributions

**Monica Canton:** conceptualization, investigation, data curation, formal analysis, visualization, and writing of the original draft, as well as review and editing. **Francesco Mirone:** investigation and visualization. **Franco Meggio:** conceptualization, investigation, and writing, including review and editing. **Alessandro Pichierri:** investigation and visualization. **Valentino Casolo:** investigation and writing, including review and editing. **Giovanni Battista Tornielli:** visualization and editing. **Andrea Pitacco:** funding acquisition, supervision, and conceptualization.

## Conflicts of Interest

The authors declare no conflicts of interest.

## Supporting information


**Figure S1:** Daily pattern of relative sap flow rates throughout the experiment, showing the average of three replicates per treatment.
**Figure S2:** Daily stomatal conductance (*g*_*s*_), in watered (green line), moderate (blue line), and severe (red line) grapevine plants.
**Figure S3:** Pictures from the grapevines under water stress.

**Table S1:** Hourly meteorological data recorded under outdoor conditions during June and July, including air temperature (°C), precipitation (mm), relative humidity (RH, %), and global radiation (W m^−2^).

## Data Availability

The data that support the findings of this study are available upon request.
