# Supplementary material for: Consecutive Moderate and Severe Drought Stresses Affect Chlorophyll Fluorescence and Non‐structural Carbohydrates Dynamics in Grapevine Leaves
Source: Physiol Plant. 2025 Sep 21;177(5):e70535. doi: 10.1111/ppl.70535 (PMC12451097; doi:10.1111/ppl.70535)
Supplement: Supplementary file 1 — Figure S1: Daily pattern of relative sap flow rates throughout the experiment, showing the average of three replicates per treatment. Figure S2: Daily stomatal conductance (g s ), in watered (green line), moderate (blue line), and severe (red line) grapevine plants. Figure S3: Pictures from the grapevines under water stress. [file PPL-177-e70535-s001.docx]

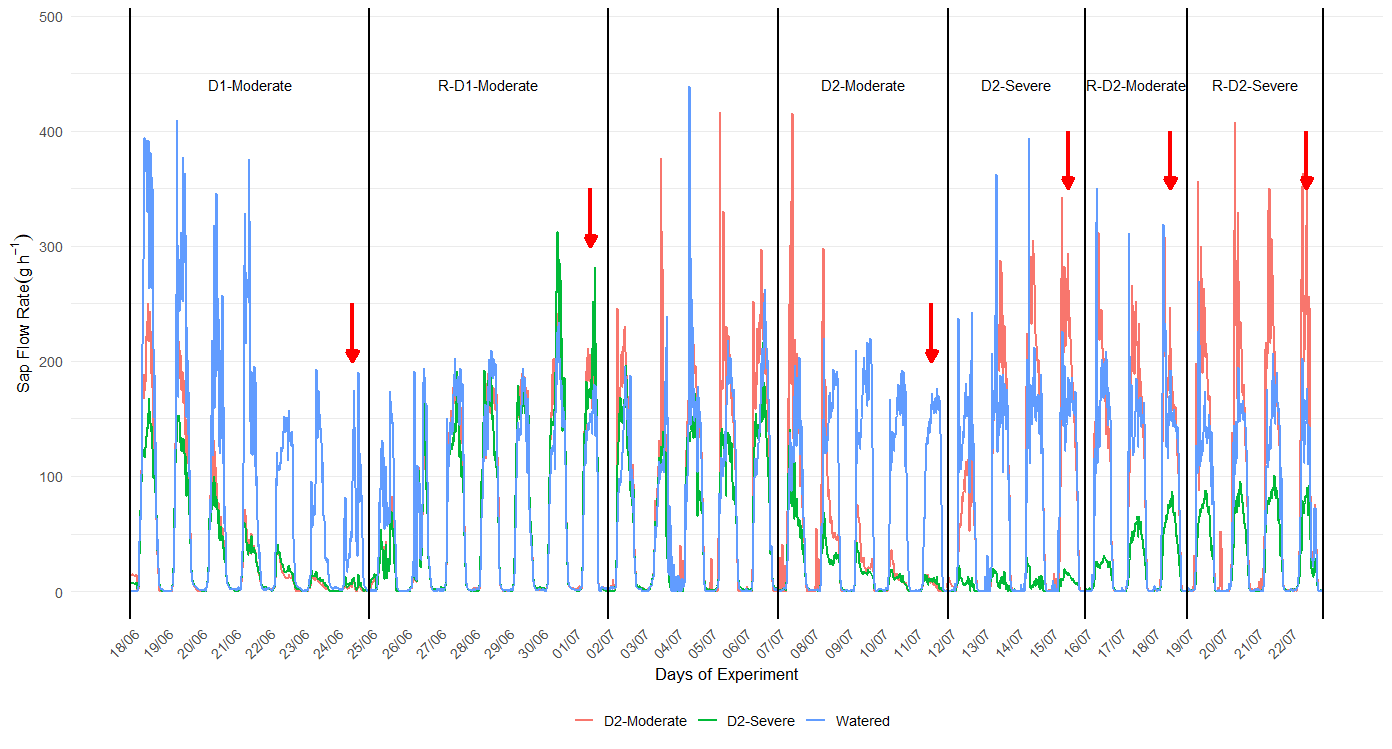


**Supplementary Figure S1.** Daily pattern of relative sap flow rates throughout the experiment, showing the average of three replicates per treatment. Each colored line represents watered plants (blue), Second Moderate (D2-Moderate; red) and Severe drought plants (D2-Severe; green) during the whole experiment period. Red arrows represent the days of measurements.


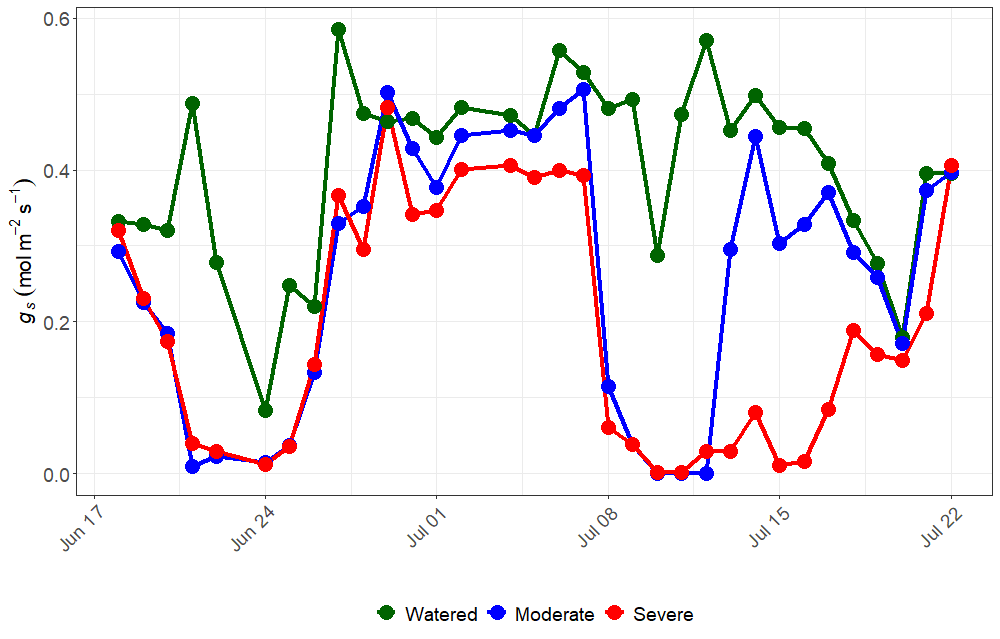


**Supplementary Figure S2.** Daily stomatal conductance (*g_s_*), in watered (green line), Moderate (blue line) and Severe (red line) grapevine plants.


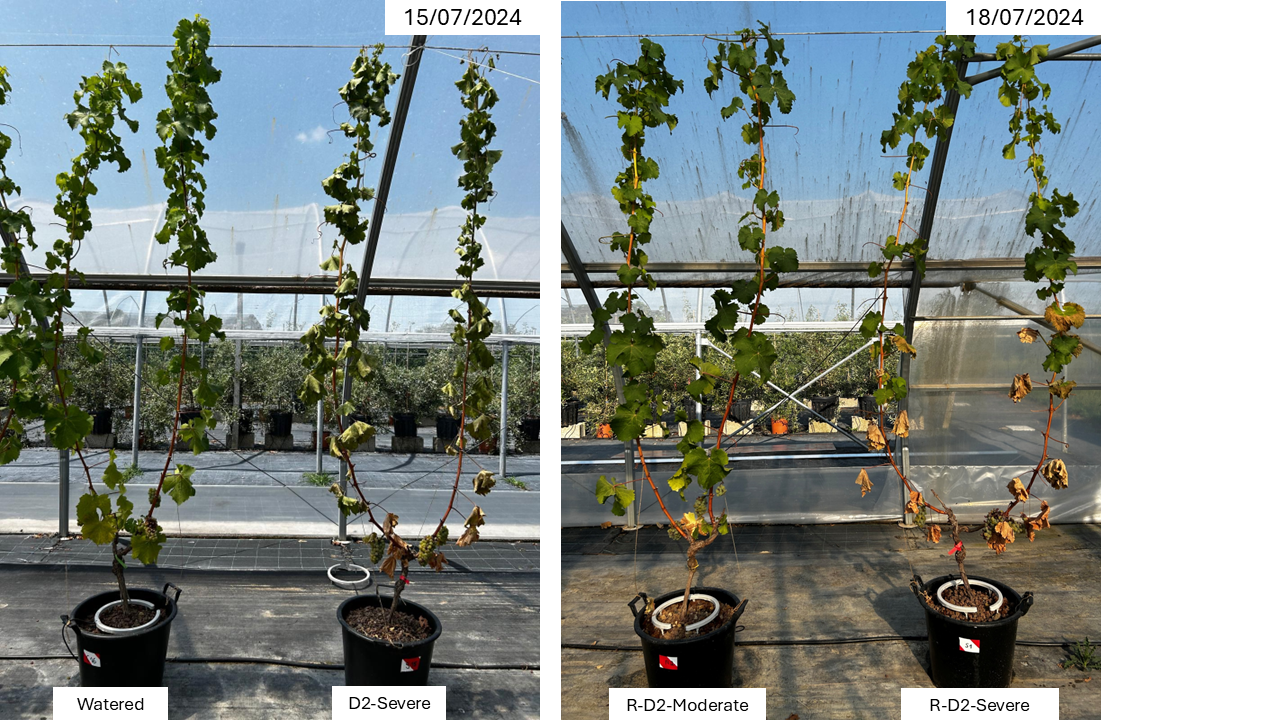


**Supplementary Figure S3.** Pictures from the grapevines under water stressed. On the left side, the photo was taken on 15/07/2025 during the D2-Severe measurements and shows the watered and D2-Severe plants. On the right side, the photo was taken on 18/07/2025 during the measurements of R-D2-Moderate and shows R-D2-Moderate and R-D2-Severe plants. On this day, R-D2-Severe was already in the third day of rewatering period and shows 50% of leaf shedding.
